# Mutation detection in women diagnosed with endometrial cancer: a next-generation sequencing analysis

**DOI:** 10.1080/23723556.2026.2692231

**Published:** 2026-06-25

**Authors:** Salar Saadi Hussain, Zahra Abdulqader Amin

**Affiliations:** a Department of Basic Sciences, College of Nursing, Hawler Medical University, Erbil, Iraq; b Department of Clinical Analysis, College of Pharmacy, Hawler Medical University, Erbil, Iraq

**Keywords:** Endometrial cancer, hereditary polyposis genes, NGS

## Abstract

Endometrial cancer (EC) is a heterogeneous gynecological malignancy characterized by diverse genetic and epigenetic alterations. This study investigated genetic mutations associated with EC among Kurdish women using next-generation sequencing (NGS). Seventy histopathologically confirmed EC cases were included, and peripheral blood DNA samples were analyzed. Whole-exome sequencing was performed on nine carefully selected cases based on specific clinical and pathological criteria, including early age of onset and/or family history suggestive of hereditary cancer predisposition, following enzymatic fragmentation, adapter ligation, PCR amplification, and targeted capture using biotinylated probes. The analysis identified five potentially significant variants in five genes: CHEK2, MUTYH, PLA2G2A, POLE, and USF3. The detected alterations included a heterozygous deletion in CHEK2 (p. Tyr113del), a homozygous SNP in MUTYH (p. Arg217His), heterozygous SNPs in PLA2G2A (p. Arg77Gly) and POLE (p. Ser2237Arg), and a heterozygous deletion in USF3 (p. Val576del). These findings highlight important molecular features of EC in Kurdish patients and may support future development of targeted therapeutic strategies. Further validation with larger cohorts is recommended.

## Introduction

Endometrial cancer (EC) is the most common malignancy of the female reproductive tract in developed and developing areas and represents a biologically heterogeneous disease with wide variations in clinical, pathological, and molecular characteristics.^1^ EC is a complex interaction of genetic mutations, epigenetic changes, hormonal regulation abnormalities, and mismatch repair (MMR) pathway defects, along with dysregulation of tumor suppressor genes and oncogenes.^2^ The evolution of molecular profiling has markedly enhanced the understanding of EC pathogenic mechanisms and ushered in a new era of personalized diagnostic/therapeutic modalities.^3^

Most EC cases are diagnosed in the early stage with a good prognosis, but 10%–15% of patients present at advanced stages or with recurrence and poor clinical outcome.^4^ The worldwide incidence of EC has become more prevalent over the past few decades due primarily to increasing rates of metabolic risk factors, including obesity, diabetes mellitus, and hypertension.^5^ Although the incidence rate is growing, mortality is relatively modest in comparison to other gynecological malignancies due primarily to the report of early-stage disease often being both detectable and treatable.^6^ However, survival outcomes differ among populations due to disparities in healthcare access, tumor biology, and genetic susceptibility.^7^

Genetic factors are critical for both the development and progression of EC. Lesions involved in tumor initiation and progression arise through both inherited and somatic mutations; they act predominantly by interfering with regulatory pathways governing cell proliferation, DNA repair, apoptosis, and genomic stability.^8^ Discrepancies in EC susceptibility have led to the identification of numerous genetic and epigenetic drivers, including susceptibility genes and several associated single-nucleotide polymorphisms (SNPs).^9^ Classifying clinically relevant genomic aberrations by therapeutic implications may facilitate early detection, enhance prognostic stratification, and inform target-directed therapies.^10^

ECs have traditionally been classified as type I or type II carcinomas based on histopathologic criteria. Type I tumors are estrogen-dependent and are usually low-grade endometrioid carcinomas with a relatively favorable prognosis.^11^ These tumors often carry mutations in genes such as *PTEN*, *KRAS*, *CTNNB1*, and *PIK3CA* and are associated with microsatellite instability.^12^ Type II tumors are estrogen-independent, with a poorly differentiated and more aggressive course associated with an adverse outcome; these tumors commonly show early changes in *TP53.* ^13^ Recently, advances in genomic technologies have shown that sole reliance on histopathological features is inadequate for the classification of EC, underlining the necessity of molecular-based characterization.^14^

The advent of next-generation sequencing (NGS) has provided a powerful high-throughput technology platform for the detection of genetic alterations driving cancer.^15^ NGS enables robust, sensitive, and high-throughput simultaneous analysis of multiple genes compared to conventional sequencing approaches.^16^ This technology is extensively used in cancer genomics studies for the identification of germline and somatic mutations, characterization of molecular pathways associated with tumorigenesis, and the potential for precision medicine approaches.^16^ NGS-based methods have enabled the identification of these clinically relevant mutations contributing to disease susceptibility and progression in EC research.^17^

Despite the major progress in genomic investigations of EC, most publicly available datasets are comprised primarily of European, North American, and East Asian populations, whereas Middle Eastern populations have yet to be adequately represented in global genomic databases, including gnomAD and The Cancer Genome Atlas (TCGA).^18^ Such under-representation restricts reliable interpretation of genetic variants, often resulting in incorrect classification of population-specific mutations. Thus, the investigation of genetic modifications in EC Kurdish women is crucial for better understanding the disease-associated variants in this unique population and improving genomic interpretation accuracy.

Overall, the current study sought to explore germline genetic variations that are correlated with EC in Kurdish women via next-generation sequencing technology, focusing on potential pathogenic variants as factors contributing to disease susceptibility and for future precision medicine opportunities.

## Methods

### Sample collection

In this case‒control study, 70 patients with EC were diagnosed based on histopathological examination, had no prior treatment (chemotherapy or radiation), and consented to participate and provide samples. Non-Kurdish women, having metastatic EC at diagnosis, and inadequate sample quality were the exclusion criteria for the current study. Socio-demographic and clinical information was obtained by direct interviews with the patients.

All samples were collected from the Maternity Teaching Hospital, Erbil, Kurdistan Region. Blood samples from patients diagnosed with EC (with a family history of EC and breast cancer and without a family history of EC and breast cancer) were taken to perform NGS, ensuring that the samples were gathered under sterile conditions to prevent contamination. All samples were immediately stored at 4°C until DNA extraction.

### DNA extraction process

Genomic DNA was extracted from whole blood EDTA samples of these patients at ExoGen Genetic Diagnostic Laboratory/Zheen International Hospital using the Pure Link™ Genomic DNA Mini Kit from Thermo Fisher, USA, following the manufacturer's protocol. The quantification and qualification of total DNA concentration were performed using the One Drop TOUCH Nano Drop Lite (Biometrics, USA) with absorbance wavelengths of the A260/A280 ratio.

### Whole-exome sequencing (WES)

Whole-exome sequencing (WES) of nine EC cases was performed using an NGS (MGI Platform, DNBSEQ-T7, China). The nine patients subjected to whole-exome sequencing (WES) were not randomly selected. They were chosen based on specific clinical and pathological criteria, including confirmed histopathological diagnosis of EC, availability of adequate high-quality DNA from blood; early age of onset (defined as diagnosis at ≤50 y of age), and/or family history suggestive of hereditary cancer predisposition and willingness to provide informed consent for genomic testing. These criteria were used to enrich the cohort for cases more likely to harbor clinically relevant germline mutations.

### Next-generation sequencing methodology

Genomic DNA samples were first quantified and then quality-checked via fluorometric methods. Library preparation was conducted using the Enzymatic Fragmentation (Twist Bioscience), involving simultaneous fragmentation and end-repair of DNA, followed by adapter ligation and PCR amplification to generate indexed libraries. The libraries then underwent hybridization-based target enrichment using the Twist Target Enrichment Standard Protocol, which utilizes biotinylated probes to selectively capture genomic regions of interest. The minimum Phred quality score was Q30, while minimum read length was 50, and bp ≥ 95% of the target bases were covered at ≥50× depth. Post-capture libraries were purified and quantified before proceeding to circularization. Using the MGIEasy Circularization Kit (MGI Tech), enriched libraries were converted into single-stranded circular DNA. Then, DNA Nanoball (DNB) generation was performed via rolling circle amplification (RCA), creating highly compact nanoballs appropriate for sequencing. Ultimately, DNBs were loaded onto MGI sequencing flow cells following the manufacturer's standard loading procedure for high-throughput sequencing.

### Bioinformatics analysis

The enriched libraries were sequenced on the MGI DNBSEQ-T7 instrument using FCL cartridges, with a target of 12.5 Gbp of data per sample, as stated by the manufacturer. Raw data analysis and comprehensive NGS data interpretation were performed using Genomize-Seq. The SEQ platform, developed by Genomize, was employed for germline variant analysis. Analyses were performed using the GRCh38/hg38 (GCA_000001405.29) genome version, and the reference version was verified prior to alignment and variant calling. The variant filtration criteria were: minimum read depth (DP) ≥ 10, minimum genotype quality (GQ) ≥ 20, and variant allele frequency (VAF) ≥ 0.2. This platform is mostly suitable for detecting hereditary genetic variants that may influence cancer predisposition, including uterine cancer. The germline pipeline incorporates industry-standard technologies for high-quality sequence alignment, functional annotation, and variant calling, facilitating comprehensive screening of cancer-related genes to identify pathogenic or possibly pathogenic variations.

### Statistical analysis

Statistical analysis was performed using the Statistical Package for Social Sciences (SPSS version 27.0 software (IBM Corp.)). Frequency and percentage were used to summarize categorical data, and mean and standard deviation were used to summarize quantitative continuous data. *p* < 0.05 was considered statistically significant. GraphPad Prism (version 10.4.1) was also used to create graphs.

## Results

### Pathway analysis of mutated genes in endometrial cancer

The demographic, clinical, and laboratory characteristics of the study participants are summarized in Table 1. The cohort exhibited a mean age of 57.20 ± 10.637 y and a BMI of 32.60 ± 7.742 kg/m², reflecting that the majority of patients were postmenopausal and overweight or obese at diagnosis. The average age at menarche was 12.73 ± 1.075 y, and that of menopause was 50.06 ± 3.766 y. In reproductive history parameters, the mean gravida and para were 4.36 ± 3.547 and 3.36 ± 2.869, respectively, with a mean abortion frequency of 1.01 ± 1.419. These clinicodemographic variables are consistent with cohort epidemiological profiles associated with increased susceptibility to EC.

**Table 1.** Demographic, clinical, and laboratory characteristics of study participants for quantitative data.

Parameters	Mean ± SD
Age (in years)	57.20 ± 10.637
BMI	32.60 ± 7.742
Age at the time of diagnosis	56.74 ± 10.783
Age at menarche	12.73 ± 1.075
Age of menopause	50.06 ± 3.766
If married, Gravida	4.36 ± 3.547
Para	3.36 ± 2.869
Abortion	1.01 ± 1.419

Detailed analysis of germline variants revealed five pathogenic or likely pathogenic variants in five cancer-associated genes: CHEK2, MUTYH, PLA2G2A, POLE and USF3 (summarized in Table 2). Out of these variants, three corresponded to missense substitutions, and two were in-frame deletion events, indicating that the mutational mechanisms contributing towards genomic instability and tumor susceptibility differed across the patient population investigated.

**Table 2.** Comprehensive genetic variant analysis of patients: genotype, pathogenic variants, and clinical associations.

Gene symbol	Quality	Ensemble gene ID	Position (hg38)	dbSNP	Alt	Ref	Nucleotide change	Gene symbol	Chromosome	Exon/Intron	Consequences
CHEK2	High	ENSG00000183765	28725346	rs1601826064	C	CAGT	CAGT->C	CHEK2	22	Exon 3	Inframe deletion
MUTYH	High	ENSG00000132781	45332445	rs140342925	T	C	C->T	MUTYH	1	Exon 9	Missense (R217H)
PLA2G2A	High	ENSG00000188257	19978078	rs779263473	C	G	G->C	PLA2G2A	1	Exon 4	Missense (R77G)
POLE	High	ENSG00000177084	132624941	rs774472240	C	G	G->C	POLE	12	Exon 48	Missense (S2237R)
USF3	High	ENSG00000176542	113659952	rs757438455	C	CCTA	CCTA->C	USF3	3	Exon 7	Inframe deletion

In particular, an in-frame deletion variant was identified in the third exon of the CHEK2 (checkpoint kinase 2) gene involved in critical pathways governing DNA damage response signalling and cell-cycle checkpoints. A missense mutation (R217H) in the base-excision repair gene MUTYH, which is critical for repairing oxidative DNA damage and maintaining genomic integrity, was identified. In addition, we identified a missense substitution (R77G) in PLA2G2A, which is involved in phospholipid metabolism and inflammatory signalling pathways that have recently been correlated with tumor progression and microenvironment modulation.

Importantly, a missense mutation in POLE exon 48 (S2237R) was also identified. The identified POLE variant (p. Ser2237Arg) is located in the polymerase domain rather than the exonuclease domain; therefore, its association with ultra-mutated EC phenotypes or high tumor mutation burden remains uncertain and requires further functional validation. Additionally, as this variant was identified from germline whole-exome sequencing of peripheral blood rather than tumor tissue, direct tumor mutation burden assessment was not performed in this study.

In summary, the identified germline variants reflect the role of DNA repair mechanisms, inflammatory pathways and transcriptional regulatory networks in shaping the molecular architecture of EC within this cohort. These results support that germline alterations in genes involved in the maintenance of genome stability may influence disease susceptibility and progression, as well as serve as potential biomarkers for risk stratification and applications in precision oncology.

### Germline mutation

We identified five germline variants, including their genotype characteristics and family history in EC patients. The genes involved were *CHEK2, MUTYH, PLA2G2A, POLE,* and *USF3*. *CHEK2* was heterozygous for a deletion (c.338_340del), which resulted in the loss of tyrosine at position 113 of the encoded protein (p. Tyr113del). A homozygous single-nucleotide polymorphism (SNP) of *MUTYH* was found (c.650G > A), coding for an amino acid replacement of arginine to histidine at position 217 (p. Arg217His). Also, a heterozygous SNP was found in *PLA2G2A* (c.229C > G), which resulted in an arginine-to-glycine change at residue 77 (p. Arg77Gly). *POLE* was heterozygous for the SNP (c.6711C > G), which caused a serine-to-arginine substitution at position 2237 (p. Ser2237Arg). Finally, a heterozygous deletion in *USF3* (c.1727_1729del) led to the loss of valine position 576 (p. Val576del) (Tables 3 and 4).

**Table 3.** Details of nucleotide variants identified in patients diagnosed with EC.

Genes	RefSeq transcript IDs (NM_ numbers)	Genotype	HGVSc	HGVSp	ACMG	ClinVar	Family history
*CHEK2*	NM_007194.4	HET	NM_007194.4: c.338_340del	NP_009125.1: p. Tyr113del	LP	VUS	BC (FDR)
*MUTYH*	NM_001048174.2	HOM	NM_001048174.2: c.650G>A	NP_001041639.1: p. Arg217His	VUS	P, LP	EC (FDR)
*PLA2G2A*	NM_001395463.1	HET	NM_001395463.1: c.229C>G	NP_001382392.1: p. Arg77Gly	VUS	Not provided	None
*POLE*	NM_006231.4	HET	NM_006231.4: c.6711C>G	NP_006222.2: p. Ser2237Arg	VUS	VUS	EC (FDR)
*USF3*	NM_001009899.4	HET	NM_001009899.4: c.1727_1729del	NP_001009899.3: p. Val576del	VUS	Not provided	None

BC: breast cancer, FDR: first-degree relative, EC: endometrial cancer, HET: heterozygous, HOM: homozygous, LP: likely pathogenic, VUS: variant of uncertain significance, P: pathogenic.

**Table 4.** Genetic variants: coverage, frequency, and functional predictions (SIFT and PolyPhen-2).

Gene symbol	Coverage(F/R)	Frequency	SIFT	PolyPhen2
*CHEK2*	42(55%)/50(54%)	0	–	–
*MUTYH*	80(100%)/67(100%)	9.00E-05	0.00 (deleterious)	1.0 (probably_damaging)
*PLA2G2A*	24(45%)/30(55%)	4.00E-05	0.17 (tolerated)	0.023 (benign)
*POLE*	34(52%)/37(49%)	0	0.05 (tolerated)	0.0 (benign)
*USF3*	44(49%)/52(57%)	1.00E-05	–	–

Such low-frequency pathogenic variants could act as population-specific biomarkers and thereby highlight unique genetic variations in the Kurdish genome. Where necessary, rare variant frequencies were checked via gnomAD, and two conservative functional predictions (SIFT and PolyPhen-2) were evaluated. In particular, MUTYH p. Arg217His was predicted to be deleterious (SIFT score 0.00, PolyPhen-2 score 1.0) and PLA2G2A p. Arg77Gly and POLE p. Ser2237Arg as tolerated or benign. The remaining variants (CHEK2 p. Tyr113del and USF3 p.Val576del) were VUS.

The sequence quality metrics showed high coverage and reliability. A mean of 105 million raw reads per sample was produced, and 95·18%–97·69% of the reads mapped to the human reference genome (Hg38). Using the Twist library kit, the targeted regions were covered on average at 99.3%, providing around 105 million reads per blood sample. The average depth of coverage in all targeted regions was 179.7× giving a very high confidence for detecting genotype variants (Table 5).

**Table 5.** Sequencing read metrics and coverage statistics.

Genes	Total reads	Mapped reads	Coverage at 50×	Average depth
*CHEK2*	101.530.388	101.521.876	97.69%	189.65
*MUTYH*	108.566.646	108.523.987	97.57%	192.75
*PLA2G2A*	96.163.252	96.152.947	96.85%	163.89
*POLE*	94.764.760	94.759.360	97.10%	165.44
*USF3*	117.421.768	117.413.654	96.93%	171.55
	143.376.778	143.345.285	97.58%	247.38
	137.400.192	137.398.639	97.56%	229.20
	68.776.352	68.753.876	95.18%	112.49
	78.338.514	78.321.650	97.31%	144.74
Mean ± SD	105148738.9 ± 24767028.7	105149030.4 ± 24777080.2	97.1 ± 0.8	179.7 ± 41.2

Overall, these findings illustrate the existence of rare germline variants with potential pathogenic implications among Kurdish women with EC. These results could serve as a foundation for population-specific genetically based screening, risk estimation and forming precision medicine approaches for this understudied population.

## Discussion

The emergence and progression of new sequencing technologies have unveiled new biological contexts in the past decade, particularly in the oncology field.^19^ In clinical settings, NGS plays a substantial role in cancer diagnosis. Until recently, tumor subtypes were classified based on their morphological characteristics; however, they are now determined primarily or entirely by genetic mutations. Researchers have found numerous cancer-associated genes using NGS, which will undoubtedly uncover other novel treatment targets.^20^

To date, over 100 cancer predisposition genes have been identified; however, the relationship between susceptibility genes and associated tumor types requires further investigation.^21^ Genetic differences played a crucial role in determining the most effective targeted therapy for individual patients, facilitating the advancement of personalized treatment approaches, including targeted therapy and immune checkpoint inhibitor (ICI) therapy, for cases of cancer recurrence or metastasis. Low-risk EC patients typically do not require further treatment; however, individuals with a specific genetic mutation may derive benefits from such interventions.^13^

This study involved whole-exome sequencing of nine patients diagnosed with EC. Five variants were identified across five genes: *CHEK2, MUTYH, PLA2G2A, POLE,* and *USF3*. These variants result in deletions or alterations to the amino acid sequence, potentially influencing gene expression and impacting protein function and stability. The current findings indicated a likely pathogenic variant (LP) based on the ACNG criteria and a variant of uncertain significance (VUS) according to ClinVar in the heterozygous *CHEK2* gene. This gene encodes a serine/threonine protein kinase that plays a critical role in regulating DNA repair, the cell cycle, and apoptosis.^22^ Numerous studies have examined the relationship between germline *CHEK2* variants and cancer predisposition, encompassing both individual case reports and extensive case‒control studies.^23^ This variant has been reported in a study concerning *CHEK2*-related cancer predisposition. There is substantial evidence indicating that deleterious germline variants in *CHEK2* heterozygotes are linked to an increased risk of female breast and prostate cancers. However, claims of elevated risks for other cancers, such as kidney, bladder, colorectal, lymphoma/leukaemia, and thyroid, were supported by minimal, biased, or conflicting evidence.^24^ Concerning the germline *CHEK2* variation on EC, few studies had evaluated its impact. A study investigated the association between EC and *CHEK2* and involved 629 colorectal cancer patients from the Netherlands, demonstrating that the c.1100delC (p.Thr367Metfs*15) variant was more frequently observed in individuals with a family history of endometrial or colorectal cancer compared to those with sporadic EC. The findings were not corroborated in a Swedish case–control study involving 705 EC cases (across all subtypes) and 1565 controls, which indicated that *CHEK2* c.1100delC was not associated with EC risk.^25^ Variants of *CHEK2* and *MUTYH* were the most prevalent, aligning with the frequencies observed in the general population.^26^ Another study found *CHEK2* missense mutation c.470T > C (p.Ile157Thr) in two patients with EC, which was associated with a minor elevated risk of breast and colon cancer.^27^

The current study identified another participant with a VUS according to ACMG, and a variant likely pathogenic or pathogenic according to ClinVar, in the homozygous (biallelic) *MUTYH* gene. The MUTYH protein in humans was regarded as a cellular protective agent that mitigates oxidative damage. MUTYH is a DNA glycosylase that facilitates the repair of post-replicative mispairs in double-stranded DNA by specifically identifying and excising adenine or 2-hydroxyadenines that are incorrectly incorporated in pairs with 7,8-dihydro-8-oxoguanine (8-oxoG), leading to G:C to T:A transversions due to errors in DNA replication or recombination (Curia et al., 2020).^28^ The patient harboring the homozygous (biallelic) MUTYH variant (p. Arg217His) was clinically evaluated for MUTYH-associated polyposis (MAP). However, no formal diagnosis of MAP was established at the time of this study, as comprehensive colonoscopy and polyposis workup data were not available for all participants. It is acknowledged that individuals with biallelic MUTYH pathogenic variants have a substantially elevated risk of developing MAP, which in turn confers an increased risk of EC. Therefore, further clinical follow-up and colorectal surveillance are strongly recommended for this patient. A previous study identified two individuals with pathogenic mutations in the *MUTYH* gene, and only one variant was bi-allelic, regarded as deleterious with a base change (c.934-2A > G).^21^

Zheng et al. (2025) conducted a study comparing the germline mutational spectra of specific HDR and MMR genes in BC and EC patients in Kazakhstan. Both gene sets displayed mutations in a substantial proportion of both cancers, supporting the notion that their tissue specificity may be more extensive than previously thought. They identified three patients with missense variations classified as pathogenic in ClinVar (two in *MUTYH* and one in MSH6). Significantly, one individual exhibited compound heterozygosity for two *MUTYH* mutations (p.G368D and p.Y151C). In a comparison of pathogenic variants in MMR genes among breast cancer patients within the same population, three patients exhibited heterozygosity for a non-pathogenic missense variant in *MUTYH*, whereas one pathogenic variant in the *MUTYH* gene (c.452A > G) (p. Tyr151Cys) was shown to be comparable between breast cancer and EC. In one study, *MUTYH* heterozygous pathogenic variants were found in eight individuals; four of these patients had the c.1187 G > A p. Gly396Asp variant, three individuals had the c.536 A > Gp. Tyr179Cys variant, and one individual had the c.1437_1439del p. Glu480del variant. In addition, they observed that c. 536 A > G p. Tyr179Cys and c.1187 G > A p. Gly396Asp were the two most common pathogenic germinal variations found in the patients, with three and four cases, respectively. These variations were quite prevalent in the overall healthy European population, occurring at frequencies of approximately 0.15% and 0.3%, respectively.^29^ The findings of a recent study conducted by Thompson et al. (2022) indicated no correlation between endometrial, colorectal, or breast cancer and *MUTYH* heterozygosity in persons of European descent.^30^ Studies indicated that heterozygotes for *MUTYH* mutations might present an approximately two-fold elevated risk for EC.^8^ Inactivation of *MUTYH* may lead to oxidative DNA damage, potentially influencing cancer etiology in various organs. A *MUTYH* variant deemed pathogenic for MAP was most likely prevalent in the tumor of a suspected familial EC case, which exhibited a tumor mutational signature aligned with the driver status of the *MUTYH* variant.^31^The current study also found an individual with a VUS according to ACMG guidelines, located in the heterozygous *PLA2G2A* gene. No previous studies exist concerning the impact of this variant on EC associated with this gene. Enzymes belonging to the tiny family of lipolytic hydrolases known as phospholipase A2 (PLA2) were found to be able to control immunological responses by producing arachidonic acid (AA), an intermediate molecule in the biosynthesis of lipid mediators such as leukotrienes, prostaglandins, etc. Secretory phospholipase A2 Group IIA (*PLA2G2A*), a member of the PLA2 family, has been linked to many malignancies, including prostate cancer.^32^

Zhang et al. (2022) reported that elevated *PLA2G2A* expression, induced by oncogenic K-ras, enhances cancer cell survival, presumably by diminishing lipid peroxidation via its role in facilitating the extraction of polyunsaturated fatty acids from lipid membranes, thereby augmenting de novo fatty acid synthesis and energy metabolism in order to support the proliferation of cancer cells. Consequently, PLA2G2A might serve as a downstream modulator of K-ras and could represent a prospective therapeutic target. Küry et al. (2008) examined various established *PLA2G2A* gene variations. The homozygous and heterozygous genotypes were analyzed and compared to the wild-type, both individually and collectively. These findings suggest that this variant is weakly to moderately linked with colorectal cancer risk. The c.-859C>G variant exhibited a protective effect. The odds ratios were 0.50, 0.82, and 0.80, suggesting a weak link with reduced colorectal cancer risk.^33^ In addition, the current study found an individual with a VUS, classified according to ACMG and ClinVar, in the heterozygous *POLE* gene. The polymerase epsilon (POLE) encodes the catalytic component of DNA polymerase epsilon, which is vital for chromosomal DNA replication and DNA repair. Abnormal gene expression and *POLE* mutations have been linked to colorectal cancer, resulting in immunodeficiency, facial dysmorphism, and reduced stature.^34^ A study identified that pathogenic *POLE* mutations were correlated with clinical advantages to immune checkpoint inhibitors (ICI), and the *POLE* gene identified in uterine cancer is likely benign, exhibiting a cDNA alteration (c.2090C>G) with a missense variant found by an NGS panel (STGA-DNA 2018).^35^ In a study of 379 EC cases analyzed via Sanger sequencing, four variations (1%, 4/379) were identified, with two located in the POLE (0.53%) and two in the POLD1 (0.53%) proofreading domains, classified as of uncertain significance according to the ACMG/AMP 2015 guidelines. Subsequent analysis employing in silico pathogenicity prediction methods indicated that all four mutations were pathogenic: c.1403A > G; p.468Y > C and c.940T > G; p.314S > A in the POLE gene, as well as c.1120G > A; p.374E > K and c.1231C > T; p.411Q > X in the *POLD1* gene.

These studies verified the concept that pathogenic germline mutations in the exonuclease proofreading domains of *POLE* (exons 9–14) and *POLD1* (exons 8–13) compromise polymerase proofreading function and predispose individuals to EC. The following likely pathogenic *POLD1* variants (c.947A>G (p.Asp316Gly); c.1433G>A (p.Ser478Asn); c.1421 T>C (p.Leu474Pro)) were identified in nine individuals with EC (from four families), as well as two likely pathogenic *POLE* variants (c.1421 T>C (p.Leu424Val); c.1089C>A (p.Asn363Lys)) in three EC individuals (from two families).^25^ In a study examining a subgroup of 50 patients (24 males and 26 females) with pancreatic ductal adenocarcinoma (PDAC), 12 (24.0%) of them were diagnosed with a pathogenic germline variant (PGV), comprising 12.0% high-risk variants and 10.0% intermediate- and low-risk variants. Another 12 patients (24.0%) were exclusively diagnosed with VUS. This included one female patient with a *POLE* gene variant (c.4523G>A) and one male patient with a gene variant (c.861T>A), both of which were classified as VUS. In the colorectal cancer (CRC) subgroup, 83 patients (41 males and 42 females) underwent germline testing, with 14 patients (16.9%) testing positive for pharmacogenomic variants (PGV), comprising 8.3% high-risk variants and 8.3% intermediate- and low-risk variants. Among the 22 patients (26.5%), only VUS were identified. Four male patients had *POLE* mutations with the variants (c.6019G>A), (c.1583C>T), (c.5650A>G), and (c.6019G>A), all of which are classified as VUS.^36^ The current study also found another participant with a VUS USF3 heterozygous gene.

*USF3*, also recognized as KIAA2018, was a transcription factor mostly expressed in the liver and skeletal muscle and is characterized by a highly conserved basic helix-loop-helix leucine zipper DNA-binding domain. Recent research has clarified the role of *USF3* in the pathogenesis of thyroid cancer and osteoporosis.^37^ The USF family comprises three members: *USF1, USF2,* and *USF3 (KIAA2018)*. The initial two family members, *USF1* and *USF2*, were widely acknowledged as USFs and have been thoroughly researched.^38^ In a study, *USF3* as a novel susceptibility gene for osteoporosis had been identified by genome-wide association studies (GWAS). Nevertheless, the functional significance of the basic helix-loop-helix transcription factor USF3 in bone metabolism and its target gene remain ambiguous. Thus, they stated that in cultured human osteoblast-like U-2OS cells, *USF3* promotes osteoblast differentiation and inhibits osteoclast genesis.^39^

Other studies documented variants in the *USF3* gene in Cowden syndrome in the absence of *PTEN* mutations.^40^ ^,^ ^41^ In a multi-generational family exhibiting Cowden syndrome-like characteristics with papillary thyroid carcinoma (PTC), a compound heterozygous germline deletion within the *USF3* gene was identified, which was present in up to 29% of unrelated individuals with Cowden syndrome or similar phenotypes and 27% of those with apparently sporadic thyroid cancer.^42^ Recent evidence suggests that molecular classification is increasingly shaping fertility-sparing strategies for young women with early-stage EC. Traditionally, candidacy for conservative management relies on histopathological criteria such as grade 1 endometrioid histology and disease confined to the endometrium. However, TCGA-based molecular subtyping is now being incorporated to refine risk assessment and ensure oncologic safety. Emerging data, including findings from a recent study,^43^ demonstrate that tumors with POLE- or MMR-deficient profiles may respond favorably to progestin-based therapy, whereas p53-abnormal and copy-number-high tumors carry a substantially higher risk of progression and are generally unsuitable for fertility-sparing approaches. These insights underscore the evolving role of molecular testing not only in prognosis and treatment stratification but also in optimizing reproductive counselling and individualized management for young patients.^44^

The genomic landscape of EC has been comprehensively characterized by The Cancer Genome Atlas (TCGA), which identified four major molecular subgroups: POLE-ultramutated, microsatellite instability (MSI) hypermutated, copy-number low (endometrioid), and copy-number high (serous-like). These classifications have important clinical implications, including prognosis and treatment sensitivity. When comparing the findings of the present study with TCGA data, the identification of a *POLE* variant—although classified as a VUS—aligns with the known relevance of POLE proofreading domain defects in the ultra-mutated subgroup, which is associated with high tumor mutational burden and a strong response to immunotherapy. Likewise, variants detected in MUTYH and CHEK2 are consistent with genes involved in DNA repair pathways that are frequently altered in the MSI-hypermutated or copy-number low groups. While TCGA datasets did not highlight PLA2G2A or USF3 as recurrent drivers, the detection of rare or population-specific variants in these genes may suggest under-represented mutation frequencies in Middle Eastern populations, highlighting the potential value of expanding EC genomic datasets beyond predominantly European cohorts. To the best of our knowledge, no studies have demonstrated a relationship between the USF3 gene and EC; hence, it might be regarded as a novel candidate gene for EC. Thus, the current findings on germline mutations indicate that the USF3 heterozygous variant (c.1727_1729del, p. Val576del), classified as a VUS, may be linked with EC. However, larger studies must be examined to evaluate the risk and percentage of endometrial malignancies linked to other genes.

## Conclusion

This study identified several germline variants—including *CHEK2, MUTYH, PLA2G2A, POLE*, and *USF3*—in a small cohort of women with EC using whole-exome sequencing. While these findings provide useful preliminary insights into the genetic architecture of EC in an under-represented population, the clinical significance of most identified variants remains uncertain. In particular, although the *USF3* variant observed here has not been previously reported in association with EC, the current evidence is insufficient to establish it as a novel candidate gene. Rather, this study should be regarded as hypothesis-generating, underscoring the importance of expanding genomic studies in diverse populations. Larger, multicenter studies—including functional characterization and replication analyses—are essential to determine the true contribution of these variants to EC susceptibility and to improve the interpretability of population-specific genomic findings.

## Data Availability

Data supporting this study are available from the corresponding author upon reasonable request.
